# CRR-Net: a correlation reconstruction and refinement network for deformable medical image registration

**DOI:** 10.1186/s42492-026-00222-4

**Published:** 2026-06-23

**Authors:** Bingxian Xie, Guimei Zhang, Ke Xu

**Affiliations:** 1 Key Laboratory of Jiangxi Province for Image Processing and Pattern Recognition, Nanchang Hangkong University, Nanchang, Jiangxi 330063 China; 2 Rose Lab, EEE Department, Nanyang Technology University, Singapore 639798, Singapore

**Keywords:** Deformable image registration, Super-resolution reconstruction, Local correlation modeling, Correlation reconstruction

## Abstract

Deformable image registration is a critical task in medical image analysis. However, existing learning-based methods encounter difficulties in achieving high registration accuracy under large anatomical deformations while maintaining model interpretability. To overcome these limitations, a correlation reconstruction and refinement network (CRR-Net) was introduced, representing the first framework to incorporate super-resolution reconstruction at the feature level for deformable registration. The core innovation lies in the correlation reconstruction and refinement module (CRRM), which enables precise modeling of spatial correspondences by leveraging high-resolution feature spaces. This design captures richer structural details and contextual cues while expanding the receptive field during resolution recovery, thereby improving performance in large-deformation scenarios. Integrated within a pyramid registration framework, the CRRM supports a multi-scale coarse-to-fine strategy based on local correlation modeling, ensuring consistent deformation-field prediction across scales. Model interpretability was further enhanced through a hierarchical visualization of the deformation fields, providing an intuitive quality assessment. Extensive experiments on brain and cardiac datasets demonstrated that CRR-Net outperforms state-of-the-art deformable registration approaches. For example, it achieved comparable performance on the LPBA40 dataset while using 32% fewer parameters and running 31% faster than CorrMLP, a representative high-performance method. This code is publicly available at https://github.com/miracledrumstick/CRR-Net.

## Introduction

Deformable image registration seeks the optimal nonlinear spatial transformation between the structures of interest in input image pairs, thereby enabling precise anatomical alignment under varying imaging conditions. It underpins key medical image analysis tasks, such as surgical simulation, atlas construction, and treatment efficacy assessment [1, 2]. Traditional registration methods [3–6] optimize deformations iteratively for each image pair. However, this procedure is computationally intensive and highly sensitive to hyperparameter tuning, which substantially limits its scalability to large-volume registration.

Recent advances in learning-based registration have introduced end-to-end architectures that provide markedly faster inference [2, 7]. For instance, VoxelMorph [8] employs a UNet-based model to estimate dense deformation fields, achieving significantly higher efficiency than traditional approaches. Building on this foundation, subsequent studies [9–13] have integrated large-kernel convolutions and vision transformers to enhance feature representation and long-range dependency modeling. Nevertheless, most methods still generate deformation fields only at the network output, restricting multi-scale refinement and reducing effectiveness in large-deformation scenarios.

To overcome these issues, recent studies have explored cascaded networks [14, 15] and pyramid frameworks [16–25], both of which adopt coarse-to-fine strategies. Although cascaded networks sequentially refine registration, each additional stage introduces substantial computational and parameter overheads. Pyramid frameworks improve multi-scale deformation refinement but still exhibit inconsistent deformation predictions across resolution levels, primarily owing to insufficient information propagation between the coarse and fine scales.

Inspired by ESPCN [26], our approach strengthens cross-scale correlation modeling by incorporating super-resolution reconstruction into multi-scale correlation features. The low-resolution correlation features are upsampled to higher resolutions through super-resolution reconstruction to ensure consistent deformation predictions across all scales. This multi-scale consistency is particularly vital for capturing large deformations (quantified here as displacements $> 10$ mm), where coarse-level alignment provides a robust global initialization for fine-level structural refinement. Beyond enforcing scale consistency, this process enriches structural detail, enhances the contextual representation, and expands the receptive field during resolution recovery, thereby improving deformation-field prediction accuracy at all scales.

This study proposes a correlation reconstruction and refinement network (CRR-Net) for deformable image registration. CRR-Net integrates a correlation reconstruction and refinement module (CRRM) that first computes local correlation feature maps between fixed and moving features through a correlation layer. The feature reconstruction block (FRB) then performs super-resolution reconstruction to produce more informative correlation representations in high-resolution feature spaces. An attention mechanism subsequently refines these representations, enhancing registration accuracy. For multi-scale fine registration, the CRRM is embedded within a pyramid architecture that enforces deformation-field consistency across scales through a coarse-to-fine process. To further improve interpretability, a hierarchical visualization of the deformation fields was introduced to depict the deformation magnitudes at different scales. The main contributions of this study are summarized as follows:

- A new network architecture is introduced, CRR-Net, incorporating CRRM as the first feature-level super-resolution reconstruction module for image registration, which enhances the correlation feature representation.
- An optimal coarse-to-fine registration strategy is developed by embedding the CRRM into a pyramid framework to ensure a consistent deformation field prediction across scales.
- A hierarchical visualization of deformation fields is provided to present the deformation magnitudes at different scales, offering an intuitive quality assessment and improved interpretability.

Experiments on three public datasets involving two registration tasks, namely, inter-patient 3D brain image registration and intra-patient 4D cardiac image registration, demonstrated the effectiveness of the proposed method.

### Deformable medical image registration

VoxelMorph [8] is a general image registration framework based on a convolutional neural network (CNN). It employs a UNet-based encoder-decoder architecture to estimate the deformation fields at the end of the decoding phase, after which a spatial transformer network [27] is applied to generate the warped image. This method demonstrated the effectiveness of unsupervised learning for paired image registration and established a foundation for subsequent research. To further enhance performance, large-kernel convolutions [10] and vision transformers [9, 11–13] have been introduced to strengthen long-range feature dependency modeling. For example, LKU-Net [10] incorporates large-kernel convolutions into the encoder, whereas TransMorph [13] integrates Swin Transformer blocks [28] into the encoder. Both approaches improve long-range feature modeling; however, their effectiveness decreases in scenarios involving large deformations.

To address the challenges posed by large and complex deformations, recent studies predominantly adopted cascaded networks [14, 15] or pyramid frameworks [16–25] for coarse-to-fine image registration. In cascaded networks, registration is performed progressively, with alignment refined stage-by-stage. For instance, LapIRN [15] cascades multiple Laplacian pyramid networks across stages, progressively optimizing the deformation field through multi-stage registration. Pyramid methods focus on hierarchical multi-scale feature optimization. For example, Im2grid [17] embeds coordinate information into multi-scale feature maps and estimates scale flows by computing neighborhood matching scores, whereas RDP [21] performs stepwise recursion at each resolution level, combining multi-scale flows to improve adaptation to complex large deformations. Despite their effectiveness, cascaded networks incur significant parameter growth and computational overheads as the number of stages increases. Pyramid methods rely on rigid-scale divisions and often suffer from inconsistent deformation predictions caused by the limited information flow between the coarse and fine scales.

To establish correspondences between the features of moving and fixed images more accurately, several studies [16, 20, 23, 24] have enhanced registration performance through explicit local correlation modeling. For example, PR++ [16] introduced a local 3D correlation layer into a dual-stream pyramid architecture to improve deformable registration. However, simple CNN architectures struggle to effectively model spatial correspondences. ModeT [20] leverages neighborhood attention to capture local correlations and estimate multi-head, multi-scale deformations; however, its small embedding dimension limits the representational capacity of its correlation features. CorrMLP [23] employed a multi-window MLP to capture long-range dependencies within local correlation features at the expense of a substantial computational cost. CGNet [24] computes global and local correlation feature maps using matrix multiplication between queries and keys within the self-attention mechanism; however, this introduces a large number of learnable parameters and increases the model complexity. Although these methods have made progress, most local correlation strategies operate on a single scale and fail to ensure consistent deformation predictions across different scales.

Although these existing methods have made significant progress, they inherently compute correlations strictly at the given spatial resolution of the extracted feature maps. This fundamentally restricts their ability to capture fine structural details without substantially increasing the model complexity. By contrast, CRR-Net is the first framework to introduce super-resolution reconstruction directly at the correlation feature level. Rather than computing dense correspondences on static or downsampled feature maps, the proposed method actively upsamples and refines the correlation representations. This novel mechanism effectively recovers high-frequency anatomical details and establishes highly accurate dense correspondences at finer scales.

### Image registration based on super-resolution

Given that super-resolution reconstruction can effectively enhance the spatial resolution and enrich fine-grained feature representations, recent studies have begun exploring its integration with image registration tasks [29–32]. These efforts aimed to address the performance limitations of existing registration methods in low-resolution or detail-deficient scenarios by leveraging the complementary advantages of both tasks. For instance, literature [29] theoretically demonstrates an intrinsic relationship between image registration and super-resolution, indicating that super-resolution modeling can fundamentally improve alignment accuracy by reducing shift estimation errors. In ref. [30], a cascaded network architecture was employed in which the image resolution was first enhanced using a super-resolution network. The reconstructed image was aligned using a registration network, thereby indirectly improving registration performance.

To the best of our knowledge, although previous studies have combined super-resolution and registration at the image level, no existing work has introduced super-resolution reconstruction into feature-level alignment.

Although the proposed method is based on existing pyramid-based approaches, its primary motivation is to ensure consistent deformation-field predictions across scales, particularly for large deformation registrations. To this end, cross-scale correlation modeling is enhanced by integrating super-resolution reconstruction into multi-scale correlation features. Specifically, low-resolution correlation features are upsampled via super-resolution reconstruction to promote consistent deformation predictions across all scales.

## Methods

**Fig. 1:**
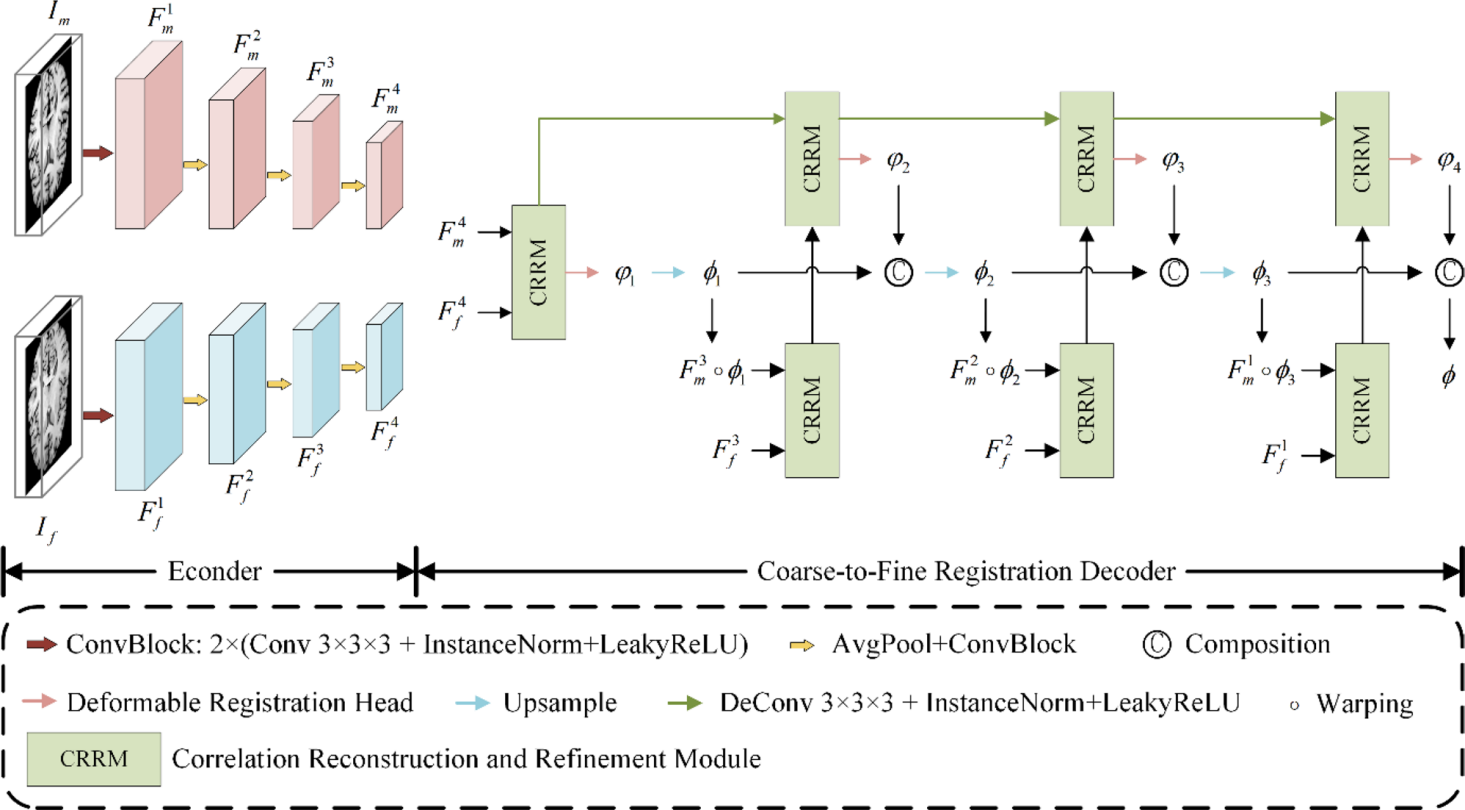
Architecture of the proposed correlation reconstruction and refinement network

The proposed CRR-Net is illustrated in Fig. 1. Given a moving image ${I_m}$ and a fixed image ${I_f}$ as input, a shared-weight encoder extracts two sets of hierarchical feature maps ${\{ F_m^i\} _{i = 1,2,3.4}}$ and ${\{ F_f^i\} _{i = 1,2,3.4}}$. The decoder consists of CRRM and a deformable registration head, which executes four coarse-to-fine registration stages. In the initial stage, the feature maps $F_m^4$ and $F_f^4$ are fed into the CRRM to generate a correlation feature map ${C_1}$. This map is then passed to the deformable registration head to predict a residual displacement field ${\varphi _1}$. From the second stage onward, two CRRMs are applied at each level to model feature-level and stage-level correlations. Specifically, the upsampled ${\varphi _1}$ (${\phi _1}$) and ${C_1}$ guide the second-stage registration, where the warped features $F_m^3 \circ \phi$ and $F_f^3$ are fed into the first CRRM. The second CRRM subsequently processes the output of the preceding CRRM together with the upsampled ${C_1}$. The resulting feature ${C_2}$ is then forwarded to the deformable registration head. A residual displacement field ${\varphi _2}$ is produced, followed by field composition with ${\phi _1}$ and upsampling to obtain the deformation sub-field ${\phi _2}$. This process is repeated twice to derive the remaining deformation sub-fields ${\phi _3}$ and ${\phi _4}$. The sub-field ${\phi _4}$ is the final deformation field $\phi$, which warps ${I_m}$ to achieve spatial alignment with ${I_f}$.

### Encoder

The encoder, which operates with shared weights, adopts a dual-stream design to independently extract hierarchical features from the input images. These features are subsequently delivered to the decoder, which contains four consecutive convolutional modules. Each module comprises convolutional layers with strides of 1 and 2. Every convolutional layer is followed by an instance normalization layer and a LeakyReLU activation function with a negative slope of 0.2. Consequently, the encoder outputs four levels of feature maps with channel dimensions of 8, 16, 32, and 64.

### CRRM

**Fig. 2:**
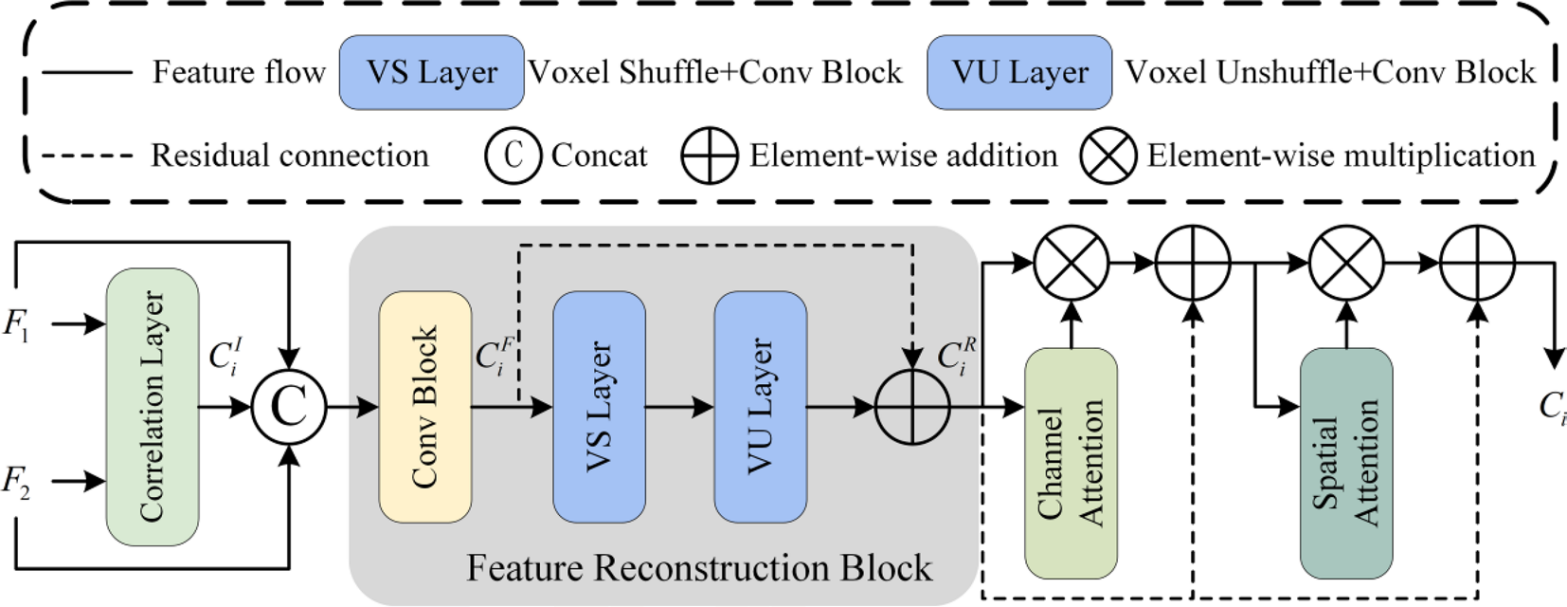
Detailed structure of the proposed correlation reconstruction and refinement module. As indicated in the legend, solid lines denote the main feature flow, while dashed lines represent residual connections used to preserve structural information

Figure 2 illustrates the architecture of the proposed CRRM, which comprises three core components: a correlation layer, a feature reconstruction block, and an attention layer. First, the correlation layer computes the correlation between each voxel of the fixed and moving features, thereby generating an initial correlation feature map. Second, the FRB reconstructs and optimizes the correlation features. Finally, channel attention and spatial attention refine the correlation feature map, thereby improving the accuracy of correspondence estimation.

First, the correlation layer computes the correlation between ${F_1} \in {\mathbb{R}^{{C_{in}} \times H \times W \times D}}$ and ${F_2} \in {\mathbb{R}^{{C_{in}} \times H \times W \times D}}$ within a local neighborhood of $k \times k \times k$. Specifically, for each spatial position $x$ of ${F_1}$, it multiplies ${F_1}\left( x \right)$ with the ${k^3}$ voxels in the local neighborhood of ${F_2}$ centered at $x$, and averages across the channel dimension to obtain a correlation feature of size ${k^3}$. This operation resembles a sliding window convolution, except that the convolution kernel is derived from the input feature maps and changes dynamically with the spatial position. Instead of summing the results, an initial correlation feature $C_1^I \in {\mathbb{R}^{{k^3} \times {C_{in}} \times H \times W \times D}}$ is obtained through feature combination, and this process contains no trainable parameters. The computation is defined as:

1 $$C_i^I\left( x \right) = {F_1}\left( x \right) \otimes {F_2}\left( {x + \delta } \right),i = 1,2,3,4$$

where $\otimes$ is the correlation calculation operation and $\delta$ is the offset relative to $x$ within the local neighborhood.

The FRB reconstructs the correlation feature map. First, the correlation features $C_i^I$, ${F_1}$, and ${F_2}$ are concatenated along the channel dimension and processed using a convolution layer for feature fusion, yielding the fused feature $C_i^F \in {\mathbb{R}^{2{C_{in}} \times H \times W \times D}}$. The VS layer then performs voxel rearrangement on the fused feature, followed by the VU layer, which restores the feature to its original resolution to produce a reconstructed feature. This reconstructed feature is then added element-wise to the initial fused feature $C_i^F$ through a residual connection to obtain the final reconstructed correlation feature $C_i^R$. Specifically, the VS layer reshapes the spatial distribution through voxel rearrangement and performs a Gaussian-filtered convolution to extract richer details. The VU layer conducts reverse voxel rearrangement using Gaussian-filtered convolution, restores spatial consistency, and expands the receptive field to capture contextual information. The voxel rearrangement operation is defined as follows:

2 $$\eqalign{& {\cal V}{\cal S}{(T)_{\left[ {c,h,w,d} \right]}} \cr& = {T_{c \cdot {r^3} + mod(h,r) \cdot{r^2} + mod(w,r) \cdot r + mod(d,r),\left\lfloor {h/r} \right\rfloor ,\left\lfloor {w/r} \right\rfloor,\left\lfloor {d/r} \right\rfloor }} \cr}$$

where $T \in {\mathbb{R}^{C{r^3} \times H \times W \times D}}$denotes the fused feature, and $C_i^F$. $C$ denotes the channel size of the rearranged output features and $r$ represents the upsampling factor, which was set to two in the experiments to construct a high-resolution feature space for refinement. $c$, $h$, $w$, and $d$ represent the coordinate indices of the output features in the channel dimensions, height, width, and depth, respectively, following voxel rearrangement. $\bmod \left( \cdot \right)$and $\left\lfloor \cdot \right\rfloor$ represent the modulo operation and floor function, respectively. The voxel-restoration operation ${\cal V}{\cal U}\left( \cdot \right)$ is mathematically defined as the inverse spatial mapping of ${\cal V}{\cal S}\left( \cdot \right)$, which aims to reconstruct the extracted high-resolution local features back to their original spatial resolutions and channel dimensions.

**Fig. 3:**
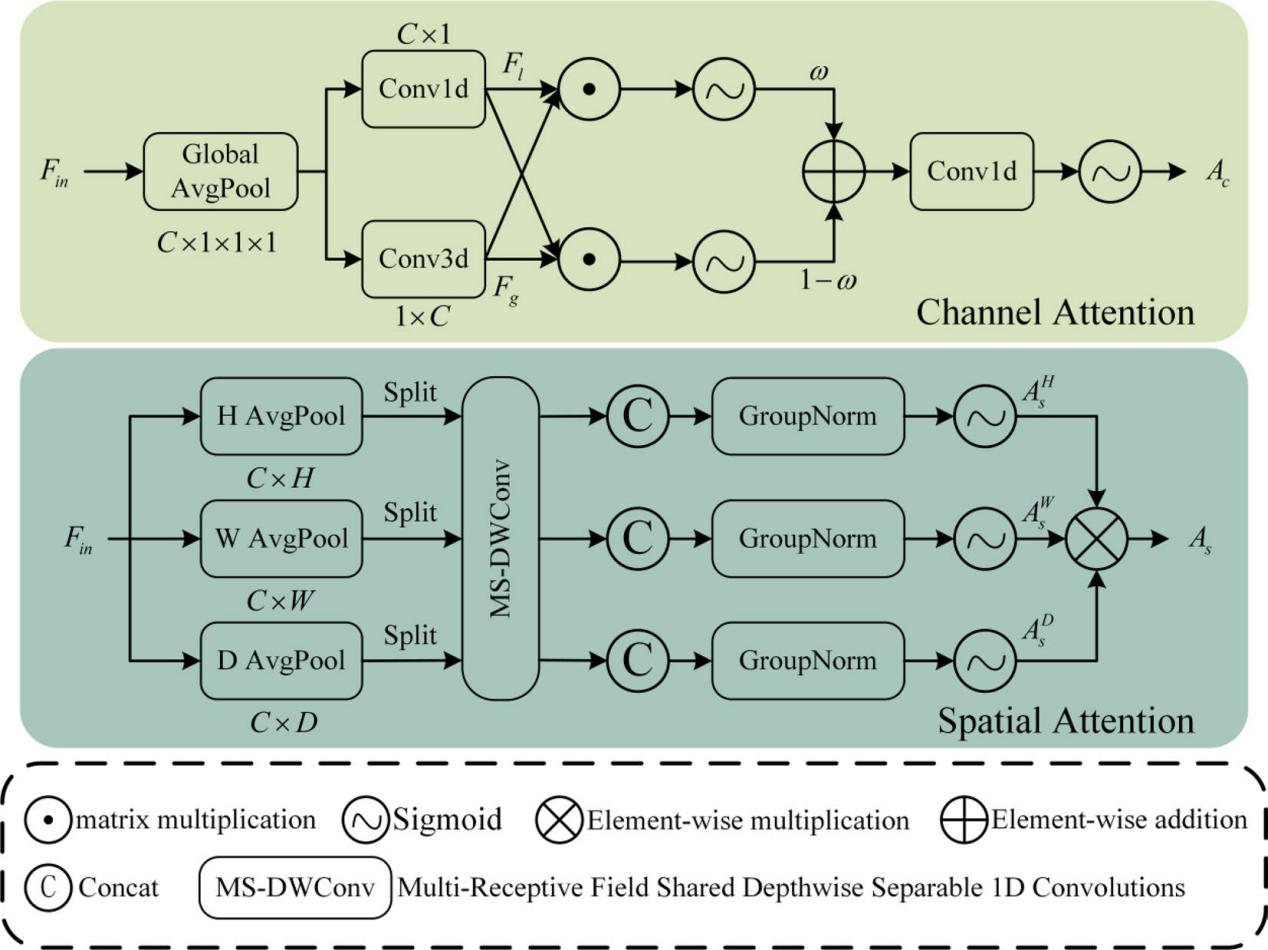
Attention layer. The module consists of channel and spatial attention layers

Inspired by previous studies [33, 34], the channel and spatial attention refine the reconstructed correlation feature $C_i^R$ and suppress inaccurate correlation responses. The detailed structures of the two attention layers are shown in Fig. 3.

In the channel attention layer, for the input feature ${F_{in}} \in {\mathbb{R}^{C \times H \times W \times D}}$, global average pooling is applied along the spatial dimensions to reduce the feature dimensionality, thereby producing the feature map ${F_{avg}} \in {\mathbb{R}^{C \times 1 \times 1 \times 1}}$. Local ${F_l} \in {\mathbb{R}^{C \times 1}}$ and global ${F_g} \in {\mathbb{R}^{1 \times C}}$ information are then processed. To strengthen the interaction between the global and local information, a cross-correlation operation is introduced to capture multi-scale correlations. A learnable factor, $\omega$ is utilized to fuse global and local information dynamically to produce the attention weight. A channel attention map is constructed using a sigmoid activation function. The computation is as follows:

3 $${A_c} = \sigma \left( {conv(\sigma (\omega \sum {{F_l}} {F_g}) + \sigma ((1 - \omega )\sum {{F_l}} {F_g}))} \right)$$

where $\sigma ( \cdot )$ is the activation function of the sigmoid.

In the spatial attention layer, for the input feature ${F_{in}} \in {\mathbb{R}^{C \times H \times W \times D}}$, average pooling is applied along height $H$, width $W$, and depth $D$ to generate one-dimensional sequence features ${F_H} \in {\mathbb{R}^{C \times H}}$, ${F_W} \in {\mathbb{R}^{C \times W}}$, and ${F_D} \in {\mathbb{R}^{C \times D}}$. These three features are each divided into four groups. Group convolutions with kernel sizes of 1, 3, 5, and 7 were applied to learn the spatial distribution and contextual relationships, while filtering redundant correlation features. The four groups are then merged, followed by group normalization and sigmoid activation to produce the attention maps $A_s^H$, $A_s^W$, and $A_s^D$. The final spatial attention map is computed as follows:

4 $${A_s} = A_s^H \otimes A_s^W \otimes A_s^D$$

where $\otimes$ is element-wise multiplication.

Specifically, to prevent the degradation of correlation features and facilitate gradient propagation during attention refinement, residual connections are employed across both the channel and spatial attention modules. Thus, the final correlation representation is defined as

5 $${C_i} = {A_s} \otimes ({A_c} \otimes C_i^R + C_i^R) + C_i^R,\quad i \in \{ 1,2,3,4\}$$

where $i \in \{ 1,2,3,4\}$ represents the hierarchical index of the decoder from coarse to fine.

### Coarse-to-fine registration strategy

To generate an accurate final deformation field, CRRM is integrated into a pyramid registration architecture, forming a coarse-to-fine registration strategy. The details are as follows. In the first stage, the low-resolution hierarchical features $F_m^4$ and $F_f^4$ extracted by the encoder are first input into the CRRM. A convolution is then adopted to predict the initial displacement field ${\varphi _0}$, after which the deformation sub-field ${\phi _1}$ is obtained through field composition and upsampling. This process is formalized as follows:

6 $${\phi _i} = {\cal U}({\phi _{i - 1}} \circ {\varphi _i} + {\varphi _i}),\quad i \in \{ 1,2,3,4\}$$

where $\circ$ denotes the warping operation, ${\cal U}\left( \cdot \right)$ represents the upsampling operation, and ${\phi _0}$ denotes an initial zero matrix.

In the second stage, the deformation sub-field ${\phi _1}$ is first employed to warp the moving feature, producing a warped feature. The warped and fixed features are then input into the first CRRM to generate a correlation feature. The resulting correlation feature and upsampled ${C_1}$ from the first stage are then sent to the second CRRM to produce the correlation feature ${C_2}$. The displacement field ${\varphi _2}$ is subsequently predicted using a deformable registration head, and ${\phi _2}$ is obtained through field composition and upsampling. This process is repeated twice to derive ${\phi _3}$ and ${\phi _4}$. The deformation sub-field ${\phi _4}$ is the final deformation field, which reaches full resolution and does not require further upsampling.

**Fig. 4:**
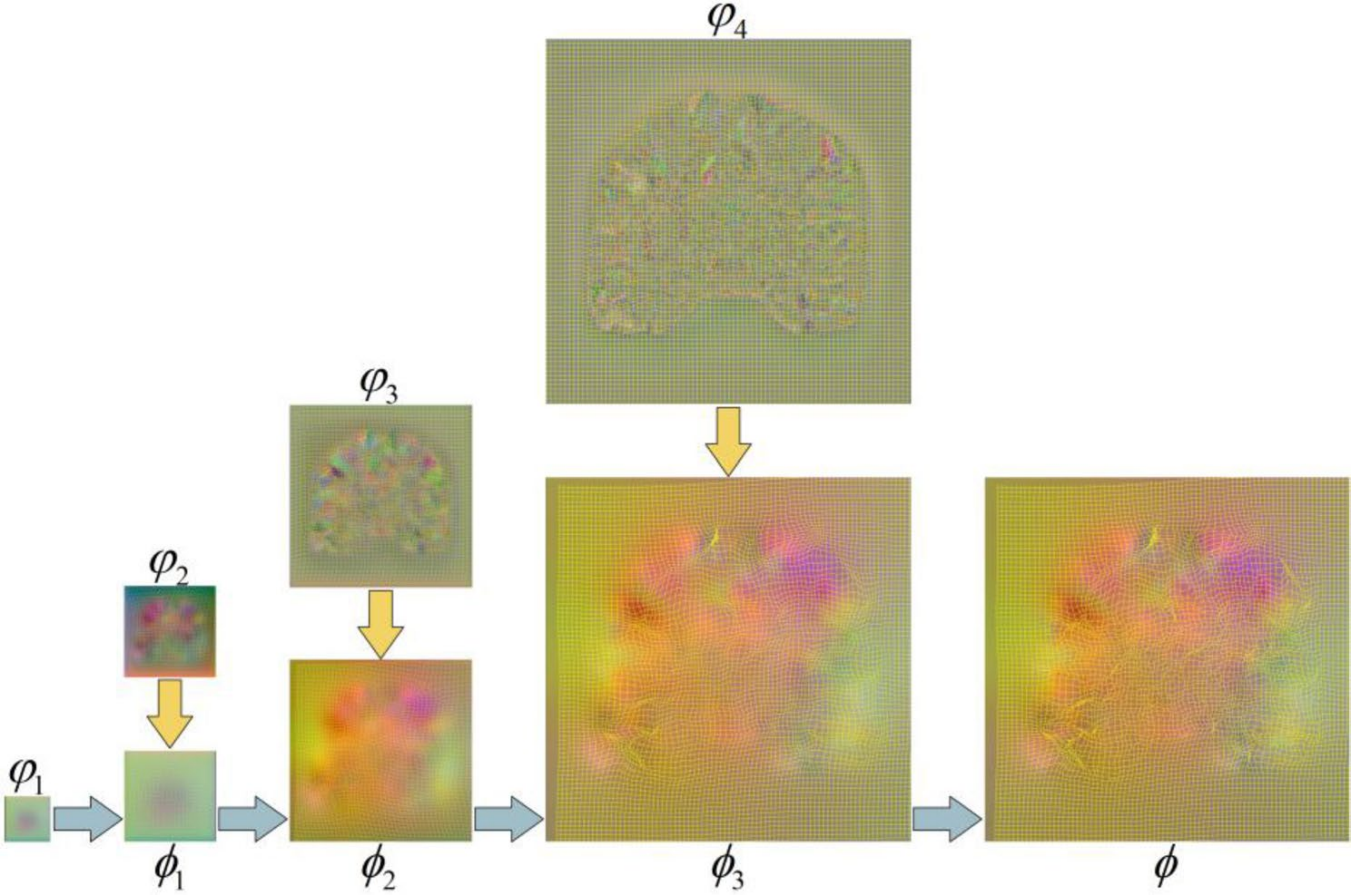
Visualization and analysis of the deformation field. Blue arrows indicate upsampling operations, and yellow arrows represent field compositions

The coarse-to-fine registration decoder integrates super-resolution reconstruction to ensure consistent deformation field prediction across coarse and fine scales. In addition, to enhance the model interpretability, the process through which CRR-Net composes the deformation field was examined (Fig. 4). As demonstrated in the visualization, the deformation field transitions from an initially smooth state focused on global large-scale alignment at lower resolutions to a progressively intricate state. This progressive incorporation of local anatomical details provides compelling visual evidence that the network effectively captures fine feature correspondences. With increasing resolution, the model actively aligns highly complex and localized anatomical structures. Consequently, the intermediate-field ${\phi _3}$ presents dense localized warping to accommodate these intricate structural differences. Upon composition with the displacement field ${\varphi _4}$, the final deformation field $\phi$ inherits complex deformations. While this demonstrates the powerful capability of the network to extract rich contextual information and enforce strict structural alignment, it inevitably introduces localized grid folding in anatomical regions with extreme spatial variations. This visual analysis underscores the fundamental mathematical trade-off between resolving fine local structural details and preserving the diffeomorphic topology during high-resolution feature reconstruction.

To guide the training, normalized cross-correlation loss ${\mathcal{L}_{ncc}}$ [35] and deformation regularization loss ${\mathcal{L}_{reg}}$ [8] are used:

7 $${\mathcal{L}_{total}} = {\mathcal{L}_{ncc}}({I_f},{I_m} \circ \phi ) + \lambda {\mathcal{L}_{reg}}(\phi )$$

where $\lambda$ is the weighting factor for the regularization term.

## Results

### Datasets

This study performed inter-patient image registration experiments on the LPBA40 [36] and Mindboggle [37] 3D brain datasets and intra-patient image registration experiments on the ACDC [38] 4D cardiac dataset.

#### LPBA40 dataset

This dataset consisted of strictly aligned images. It included 40 T1-weighted magnetic resonance imaging (MRI) images, each with 54 manually labeled regions of interest (ROIs). All MRI images underwent strict preprocessing and were aligned to the MNI305 standard space. After centering, the images were cropped to $160 \times 192 \times 160$ ($1mm \times 1mm \times 1mm$). To maximize the diversity of the anatomical variations during training and evaluation, an exhaustive pairing strategy was employed for the inter-patient registration task. Specifically, each image was paired with every other image within its respective split, alternately serving as moving and fixed images. This arrangement resulted in $30 \times 29 = 870$ training and $10 \times 9 = 90$ testing pairs.

#### Mindboggle dataset

This dataset consists of images that have been affine pre-aligned to the MNI152 space. Each image was manually labeled using 62 ROIs. All images were centered and cropped to $160 \times 192 \times 160$ ($1mm \times 1mm \times 1mm$). Among them, 42 MRI images from the NKI-RS-22 and NKI-TRT-20 subsets were used for training, and 20 MRI images from the OASIS-TRT-20 subset were used for testing. Following the identical exhaustive pairing strategy used for the LPBA40 dataset, the inter-patient combinations yielded $42 \times 41 = 1722$ pairs for training and $20 \times 19 = 380$ pairs for testing. All images in the two brain datasets underwent min-max normalization and skull stripping using FreeSurfer [39].

#### ACDC dataset

This dataset contains cardiac images, including 4D cine-MRI scans from 150 patients, with segmentation labels for the left ventricular cavity, right ventricular cavity, and myocardium. In the experiments, end-diastolic images were used as moving images, and end-systolic images were used as fixed images. All images were resampled to a voxel spacing of $1.5mm \times 1.5mm \times 3.15mm$, cropped to $128 \times 128 \times 32$, centered on the heart, and normalized to the range [0, 1] using min-max normalization. The dataset contained 100 images for training and 50 images for testing. The 100 training images were randomly split into 90 for training and 10 for validation, and the 50 test images were used for evaluation.

### Implementation details

All the experiments were conducted in a standardized hardware and software environment. The hardware configuration included a CentOS system equipped with an NVIDIA GeForce RTX 3090 GPU with 24 GB of memory, and an Intel(R) Xeon(R) Gold 5318Y CPU @ 2.10 GHz. The software environment was based on PyTorch 1.12.1, which was employed for model training and inference. The Adam optimizer was employed with a batch size of 1, an initial learning rate of 0.0001, and a regularization parameter of 1. The CRR-Net was trained for 30 epochs on the LPBA40 and Mindboggle brain datasets for deformable registration. For the ACDC cardiac image registration, CRR-Net was trained for 100 epochs.

### Evaluation metrics

To quantitatively evaluate registration performance, a set of evaluation metrics widely adopted by the medical image registration community was employed [20, 21]. Specifically, the overlap between the corresponding regions and the similarity of the region contours were evaluated using the Dice similarity coefficient (DSC), average symmetric surface distance (ASSD), and 95% Hausdorff distance (HD95). The diffeomorphism of the deformation fields was evaluated by calculating the percentage of voxels with a negative Jacobian determinant ($|{J_\phi }| < 0$). The model inference efficiency was measured using the average runtime per image pair, and the model complexity was assessed by counting the number of learnable parameters. An ideal registration model should achieve a high DSC, low ASSD and HD95 values, a low percentage of negative Jacobian voxels, and faster inference speed with fewer learnable parameters.

To assess the statistical significance, two-sided Wilcoxon signed-rank tests were performed between the proposed and each comparison method based on per-patient DSC scores.

### Comparison with existing methods

CRR-Net has been extensively compared with several state-of-the-art deformable image registration methods, including SPM, SyN [3], VoxelMorph [8], TransMorph [9], TransMatch [13], PR++ [16], PIVIT [19], Im2grid [17], NICETrans [22], ModeT [20], RDP [21], and CorrMLP [23]. For the traditional baselines, SPM was configured using its old normalise tool, and the SyN method was implemented using the ANTsPy library, with the number of iterations set to 160, 80, and 40. To ensure a fair comparison and consistency in the experimental settings, all deep learning methods were trained and evaluated using identical preprocessing procedures, spatial resolutions, dataset splits, and loss functions identical to those used in CRR-Net. In addition, all models were trained on the same GPU platform, with hyperparameters tuned to achieve optimal registration performance.

Tables 1, 2 and 3 present a quantitative comparison between CRR-Net and existing methods for brain and cardiac image registration. Among these methods, pyramid-based registration approaches achieved higher DSC scores, lower HD95 values, and lower ASSD values than direct registration methods. These results validated the advantage of pyramid structures in capturing spatial correspondences under large deformations. However, these methods involve more parameters and longer inference times, indicating increased model complexity and reduced efficiency. Notably, PIVIT achieved faster inference than direct registration methods by introducing transformer blocks only at low-resolution layers, whereas its registration performance remained inferior. Learning-based methods employ a consistent regularization loss, resulting in minimal differences in the percentage of voxels with a negative Jacobian determinant. RDP demonstrated a lower percentage of negative Jacobian voxels because of scaling and squaring operations applied to the deformation field [40].

**Table 1:** Performance comparison of different methods on the LPBA40 dataset

Method Type		DSC (%)	HD95 (mm)	ASSD (mm)	$|{J_\phi }| < 0$ (%)	$t$ (s)	Parameter (M)
Initial		53.7 ± 4.8	7.43 ± 0.78	2.77 ± 0.34	-	-	-
SPM	Traditional	68.6 ± 1.3^*^	5.69 ± 0.49	1.83 ± 0.11	-	55.35	-
SyN	Traditional	70.4 ± 1.8^*^	5.82 ± 0.50	1.75 ± 0.13	**< 0.0003**	> 10^3^	-
VoxelMorph	CNN, direct	64.5 ± 3.1^*^	6.57 ± 0.65	2.06 ± 0.22	< 0.6	0.10	**0.30**
TransMorph	Transformer, direct	68.4 ± 2.8^*^	6.27 ± 0.64	1.86 ± 0.20	< 0.5	0.20	46.77
TransMatch	Transformer, direct	67.8 ± 2.8^*^	6.25 ± 0.64	1.89 ± 0.20	< 0.4	0.24	86.80
PR++	CNN, pyramid	69.3 ± 2.3^*^	6.13 ± 0.61	1.81 ± 0.18	< 0.1	0.52	1.24
PIVIT	Transformer, pyramid	70.5 ± 1.8^*^	5.77 ± 0.53	1.73 ± 0.14	< 0.02	**0.03**	0.66
Im2grid	Transformer, pyramid	70.6 ± 1.5^*^	5.71 ± 0.50	1.69 ± 0.12	< 0.009	0.29	0.89
NICETrans	Transformer, pyramid	72.8 ± 1.4^*^	5.44 ± 0.44	1.57 ± 0.10	< 0.3	0.29	5.62
ModeT	Transformer, pyramid	72.0 ± 1.5^*^	5.55 ± 0.48	1.61 ± 0.11	< 0.005	0.56	1.03
RDP	CNN, pyramid	73.0 ± 1.4^*^	5.55 ± 0.50	1.58 ± 0.13	< 0.0007	1.02	8.92
CorrMLP	MLP, pyramid	73.4 ± 1.4^*^	5.43 ± 0.44	1.54 ± 0.10	< 0.3	0.81	4.19
CRR-Net	CNN, pyramid	**73.7 ± 1.4**	**5.38 ± 0.46**	**1.53 ± 0.10**	< 0.07	0.56	2.84

All running times were measured on the same GPU platform. Bold values indicate the best performance. *P < 0.05 compared with CRR-Net. DSC: Dice similarity coefficient; ASSD: Average symmetric surface distance; HD95: 95% Hausdorff distance; CRR-Net: Correlation reconstruction and refinement network

**Table 2:** Performance comparison of different methods on the Mindboggle dataset

Method	DSC (%)	HD95 (mm)	ASSD (mm)	$\left| {{J_\phi }} \right| < 0$**(%)**
Initial	31.8 ± 2.4	7.12 ± 0.61	2.31 ± 0.25	-
SPM	49.2 ± 1.5	5.81 ± 0.43	1.58 ± 0.11	-
SyN	56.5 ± 1.4^*^	5.69 ± 0.40	1.46 ± 0.10	**< 0.000006**
VoxelMorph	53.6 ± 2.4^*^	6.47 ± 0.56	1.69 ± 0.19	< 0.2
TransMorph	58.6 ± 2.4^*^	6.17 ± 0.55	1.53 ± 0.18	< 0.9
TransMatch	57.2 ± 2.2^*^	6.13 ± 0.55	1.54 ± 0.17	< 0.9
PR++	59.3 ± 2.0^*^	6.11 ± 0.54	1.50 ± 0.17	< 0.4
PIVIT	57.1 ± 1.5^*^	5.66 ± 0.41	1.44 ± 0.11	< 0.07
Im2grid	59.0 ± 1.6^*^	5.72 ± 0.42	1.41 ± 0.12	< 0.01
NICETrans	64.5 ± 1.3	**5.39 ± 0.38**	1.27 ± 0.10	< 0.7
ModeT	61.9 ± 1.2^*^	5.50 ± 0.35	1.32 ± 0.09	< 0.02
RDP	64.5 ± 1.4^*^	5.57 ± 0.43	1.30 ± 0.11	< 0.006
CorrMLP	**64.8 ± 1.2**	5.40 ± 0.39	**1.26 ± 0.10**	< 0.7
CRR-Net	**64.8 ± 1.2**	5.40 ± 0.39	**1.26 ± 0.10**	< 0.3

Bold values indicate the best performance. *P < 0.05 compared with CRR-Net. DSC: Dice similarity coefficient; ASSD: Average symmetric surface distance; HD95: 95% Hausdorff distance; CRR-Net: Correlation reconstruction and refinement network

**Table 3:** Performance comparison of different methods on the ACDC dataset

Method	DSC (%)	HD95 (mm)	ASSD (mm)	$\left| {{J_\phi }} \right| < 0$**(%)**
Initial	58.0 ± 10.8	5.95 ± 1.61	2.35 ± 0.77	-
SyN	79.6 ± 6.0^*^	3.20 ± 1.16	1.12 ± 0.41	**0**
VoxelMorph	74.6 ± 7.8^*^	3.66 ± 1.20	1.36 ± 0.50	< 0.05
TransMorph	77.8 ± 7.4^*^	3.42 ± 1.22	1.16 ± 0.44	< 0.8
TransMatch	77.1 ± 7.5^*^	3.41 ± 1.17	1.21 ± 0.45	< 0.1
PR++	80.4 ± 6.7^*^	3.17 ± 1.17	1.06 ± 0.43	< 0.03
PIVIT	76.5 ± 8.3^*^	3.46 ± 1.25	1.26 ± 0.52	< 0.003
Im2grid	72.1 ± 8.7^*^	3.95 ± 1.27	1.50 ± 0.56	< 0.003
NICETrans	80.8 ± 6.3^*^	2.92 ± 0.99	1.03 ± 0.39	< 0.1
ModeT	79.8 ± 6.8^*^	3.01 ± 1.07	1.08 ± 0.44	< 0.007
RDP	83.1 ± 4.8^*^	2.74 ± 0.94	0.91 ± 0.31	< 0.0001
CorrMLP	82.7 ± 5.5^*^	2.74 ± 0.92	0.92 ± 0.64	< 0.1
CRR-Net	**83.3 ± 5.2**	**2.68 ± 0.93**	**0.90 ± 0.33**	< 0.03

Bold values indicate the best performance. *P < 0.05 compared with CRR-Net. DSC: Dice similarity coefficient; ASSD: Average symmetric surface distance; HD95: 95% Hausdorff distance; CRR-Net: Correlation reconstruction and refinement network

**Fig. 5:**
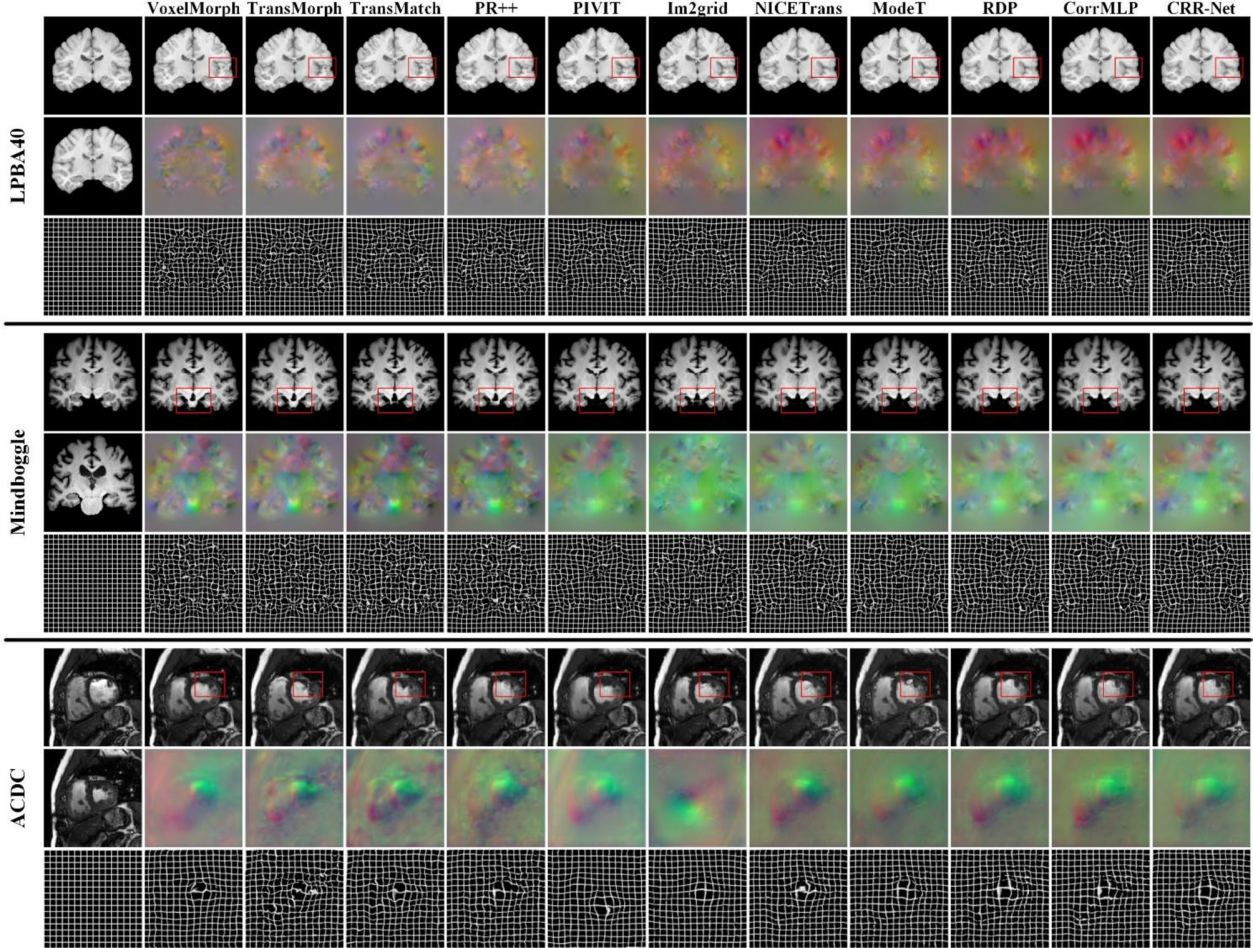
Qualitative results for LPBA40 (rows 1–3), Mindboggle (rows 4–6), and ACDC (rows 7–9). For each dataset, the first column shows a fixed image, a moving image, and a regular grid. For the remaining columns, the rows (from top to bottom) present the warped moving images, deformation fields (RGB) and deformation grids. The red boxes highlight specific local anatomical regions for a closer visual comparison of structural alignment details across different methods

Furthermore, compared to recent correlation-based approaches, such as ModeT and CorrMLP, CRR-Net demonstrated an optimal trade-off between registration accuracy and computational efficiency. Although ModeT operates with fewer parameters, its limited representational capacity results in lower DSC scores. By using multi-window MLPs within a pyramid architecture, CorrMLP achieved higher DSC scores than most competing methods but incurred substantial computational costs. Nevertheless, owing to the efficient feature-level super-resolution mechanism, CRR-Net successfully outperformed CorrMLP in terms of registration accuracy while providing significantly faster inference and fewer model parameters (Table 2). In addition, CRR-Net achieved lower HD95 and ASSD values, indicating that the resulting registration preserved anatomical boundaries more accurately. In Fig. 5, the red boxes highlight specific local regions where CRR-Net achieved more accurate structural alignment than other methods. Although the visual improvements were subtle owing to the strong performance of the baseline methods, these highlighted areas indicated that CRR-Net successfully captured more detailed feature correspondences during the registration process.

**Fig. 6:**
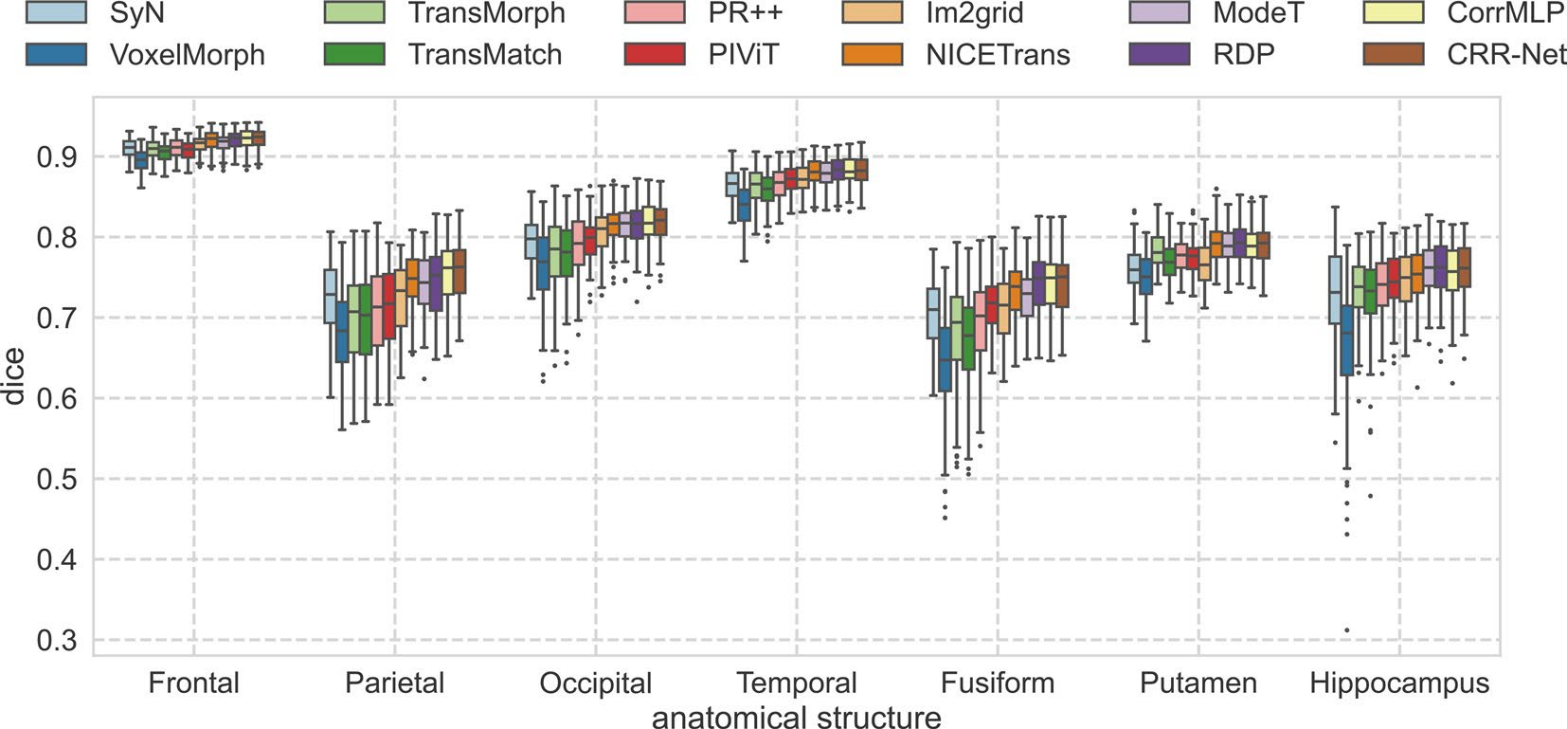
Box plots showing the distributions of Dice similarity coefficient scores for the seven regions in the LPBA40 dataset produced by different registration methods. To ensure visual clarity, representative functional regions were strategically selected, and their corresponding bilateral subregions were aggregated from the original 54 regions of interest

**Fig. 7:**
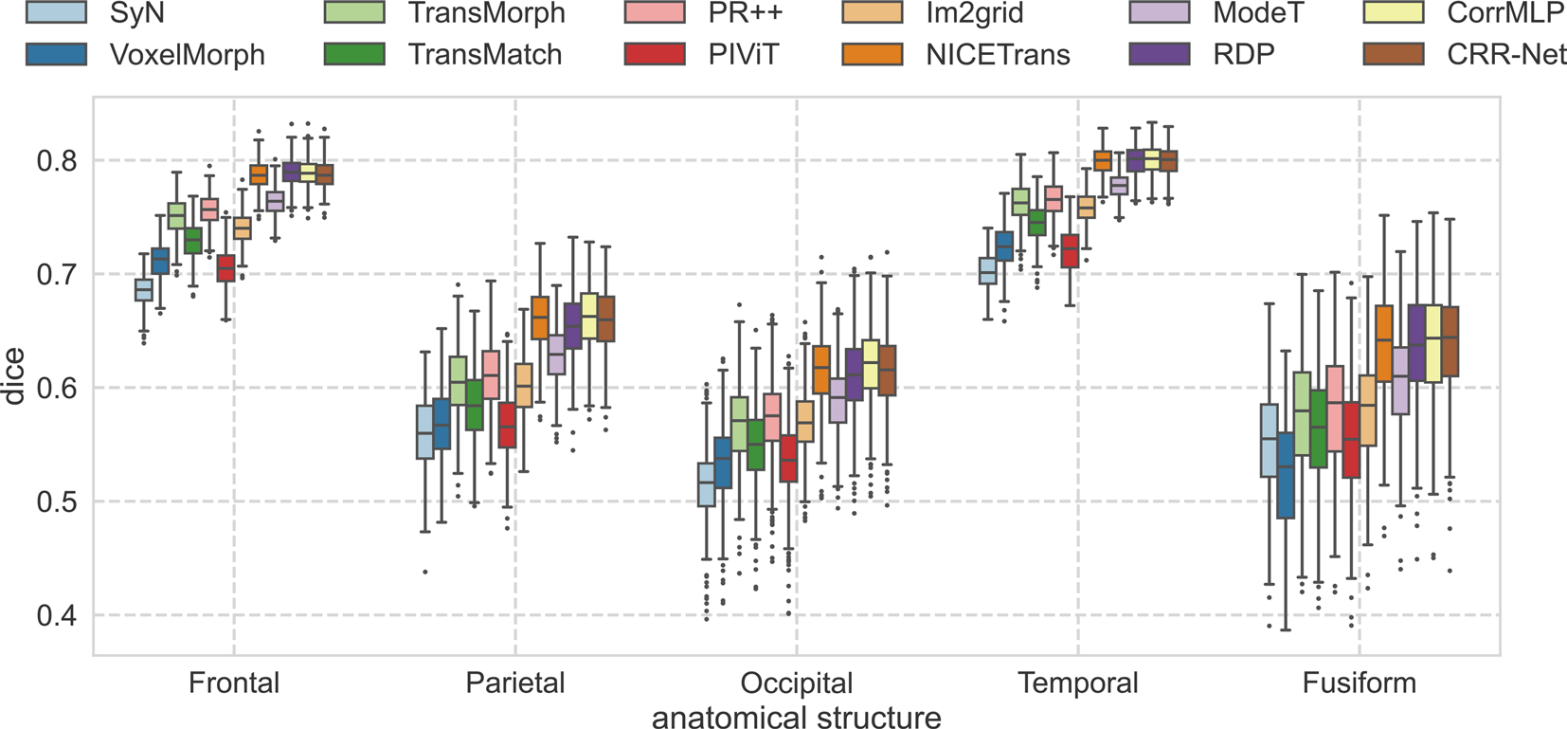
Box plots showing the distributions of Dice similarity coefficient scores for the five regions in the Mindboggle dataset produced by different registration methods. To ensure visual clarity, representative functional regions were strategically selected, and their corresponding bilateral subregions were aggregated from the original 62 regions of interest

**Fig. 8:**
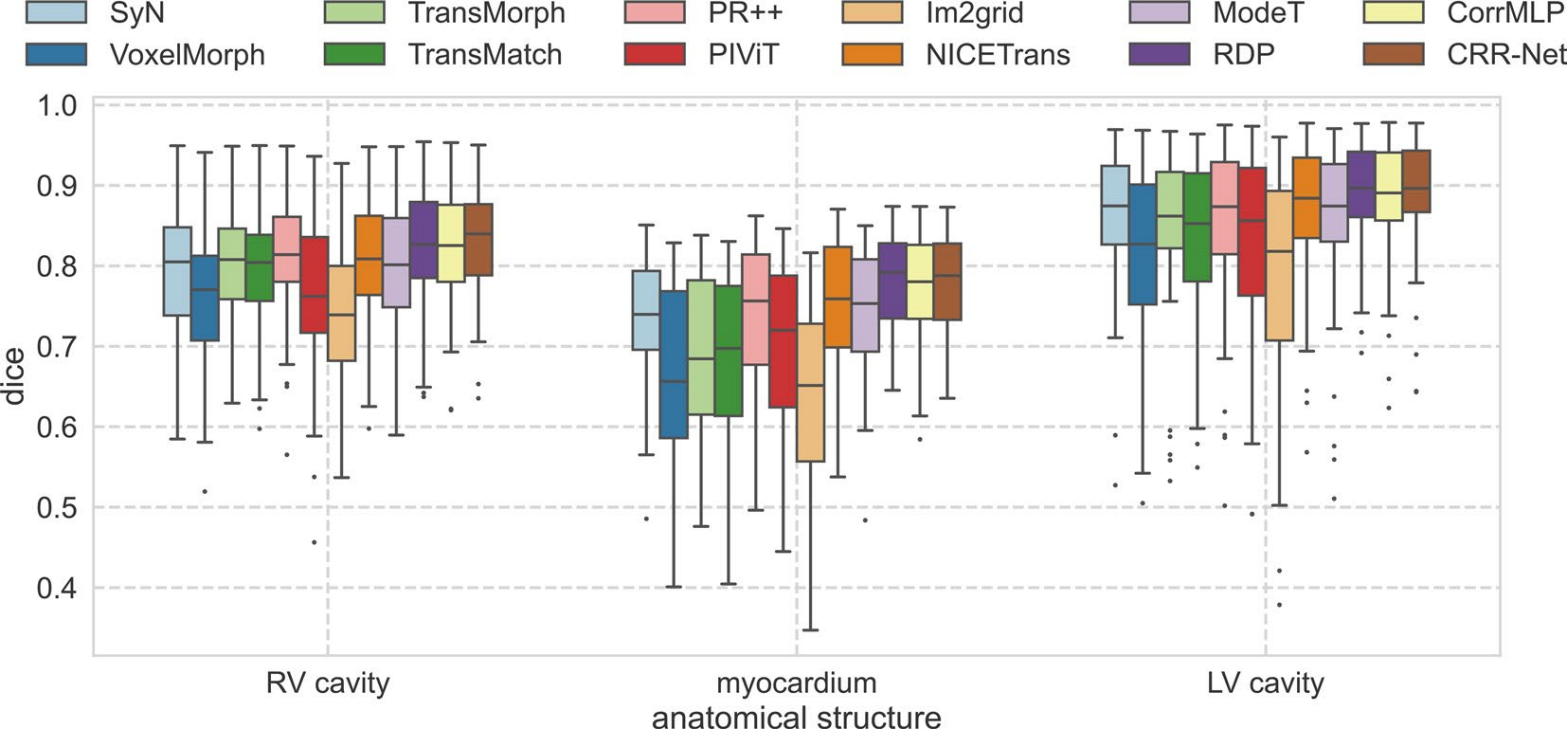
Box plots showing the distributions of Dice similarity coefficient scores for the three regions in the ACDC dataset produced by different registration methods

To further investigate the correspondence between anatomical regions across different registration methods, box plots of the selected regions in the three datasets (Figs. 6, 7 and 8) are presented. CRR-Net outperformed other state-of-the-art methods on the LPBA and ACDC datasets, demonstrating its superior capability in handling large local deformations. However, for the Mindboggle dataset, CRR-Net performed slightly worse than CorrMLP and NICETrans. This difference was attributed to the incorporation of a 7 × 7 × 7 window branch in the decoder stage by CorrMLP, which enabled it to capture deformations over larger regions, albeit at the cost of higher computational overhead. NICETrans further introduced affine pre-alignment and transformer blocks into the decoder, which increased the number of learnable parameters and model complexity.

To further evaluate the effectiveness of the proposed CRR-Net in large-deformation scenarios, a quantitative analysis was conducted by stratifying the test image pairs according to their deformation magnitudes. Specifically, the overall deformation severity of each test pair was quantified by calculating the percentage of foreground voxels with displacements exceeding 10 mm. Based on this metric, all image pairs were categorized into three severity groups: mild ($\le 1\%$), moderate ($1\% - 4\%$), and severe ($> 4\%$). These thresholds were derived from comprehensive statistical and visual analyses of the deformation distributions across the LPBA40, Mindboggle, and ACDC datasets. Empirically, setting the thresholds at 1% and 4% effectively accommodates the diverse characteristics of these datasets while preventing severe data imbalance across the stratified groups.

Using this stratification strategy, the performance of all compared methods was evaluated. As shown in Tables 4, 5 and 6, on the LPBA40 dataset, CRR-Net achieved optimal performance in the mild and moderate deformation groups and demonstrated performance comparable to that of RDP in the severe deformation group. For the Mindboggle and ACDC datasets, although the overall performance differences among the methods were relatively minor, CRR-Net consistently maintained a competitive advantage when handling moderate and severe deformations.

**Table 4:** Quantitative evaluation of registration performance under varying deformation magnitudes on the LPBA40 dataset

Method	Mild		Moderate		Severe	
	DSC (%)	$\left| {{J_\phi }} \right| < 0$**(%)**	DSC (%)	$\left| {{J_\phi }} \right| < 0$**(%)**	DSC (%)	$\left| {{J_\phi }} \right| < 0$**(%)**
Initial	53.2 ± 4.8	-	53.3 ± 5.2	-	54.3 ± 4.7	-
SPM	68.3 ± 1.5	-	68.5 ± 1.2	-	68.8 ± 1.4	-
SyN	70.4 ± 1.4	< **0.00009**	70.7 ± 1.9	**< 0.0008**	70.2 ± 1.9	**< 0.00002**
VoxelMorph	64.2 ± 2.5	< 0.6	64.1 ± 3.6	< 0.6	65.0 ± 3.1	< 0.6
TransMorph	67.6 ± 3.5	< 0.5	68.6 ± 2.2	< 0.4	69.8 ± 2.7	< 0.5
TransMatch	67.0 ± 3.4	< 0.5	68.1 ± 2.2	< 0.4	68.0 ± 2.9	< 0.4
PR++	68.5 ± 2.8	< 0.1	69.2 ± 2.1	< 0.1	69.9 ± 2.1	< 0.1
PIVIT	70.0 ± 2.3	< 0.02	70.4 ± 1.7	< 0.02	70.9 ± 1.5	< 0.02
Im2grid	70.0 ± 1.8	< 0.0009	70.6 ± 1.2	< 0.0008	71.1 ± 1.4	< 0.01
NICETrans	72.5 ± 1.8	< 0.4	72.8 ± 1.2	< 0.3	73.1 ± 1.4	< 0.3
ModeT	71.4 ± 1.8	< 0.005	71.9 ± 1.2	< 0.004	72.3 ± 1.4	< 0.005
RDP	72.5 ± 2.0	< 0.0008	72.6 ± 1.6	< 0.0007	**73.6 ± 1.4**	< 0.0007
CorrMLP	73.6 ± 1.0	< 0.3	73.3 ± 1.3	< 0.3	73.4 ± 1.8	< 0.3
CRR-Net	**74.0 ± 0.9**	< 0.07	**73.6 ± 1.2**	< 0.08	73.6 ± 1.5	< 0.08

The test pairs are divided into three severity groups: Mild ($\le 1\%$), Moderate ($1\% - 4\%$), and Severe ($> 4\%$), based on the percentage of foreground voxels with displacements greater than 10 mm. DSC: Dice similarity coefficient

**Table 5:** Quantitative evaluation of registration performance under varying deformation magnitudes on the Mindboggle dataset

Method	Mild		Moderate		Severe	
	DSC (%)	$\left| {{J_\phi }} \right| < 0$**(%)**	DSC (%)	$\left| {{J_\phi }} \right| < 0$**(%)**	DSC (%)	$\left| {{J_\phi }} \right| < 0$**(%)**
Initial	31.9 ± 2.2	-	31.6 ± 2.6	-	27.8 ± 2.9	-
SPM	49.2 ± 1.6	-	49.3 ± 1.4	-	49.2 ± 0.6	-
SyN	56.5 ± 1.4	< **0.000008**	56.5 ± 1.5	**< 0.000003**	55.6 ± 1.0	**0**
VoxelMorph	53.8 ± 2.1	< 0.6	53.4 ± 2.7	< 0.6	49.2 ± 3.9	< 0.6
TransMorph	58.3 ± 2.6	< 0.9	59.1 ± 2.0	< 0.8	59.2 ± 1.1	< 0.9
TransMatch	56.9 ± 2.4	< 0.9	57.6 ± 1.8	< 0.9	57.7 ± 1.1	< 1
PR++	60.2 ± 1.5	< 0.4	58.0 ± 1.9	< 0.5	52.8 ± 0.4	< 0.6
PIVIT	57.2 ± 1.4	< 0.06	57.0 ± 1.7	< 0.07	56.0 ± 1.2	< 0.1
Im2grid	58.9 ± 1.7	< 0.01	59.2 ± 1.4	< 0.01	58.5 ± 1.6	< 0.02
NICETrans	64.5 ± 1.3	< 0.7	64.5 ± 1.2	< 0.8	64.1 ± 1.1	< 0.8
ModeT	61.9 ± 1.2	< 0.02	61.9 ± 1.1	< 0.02	60.5 ± 1.8	< 0.03
RDP	64.4 ± 1.4	< 0.006	64.5 ± 1.3	< 0.006	63.8 ± 1.3	< 0.007
CorrMLP	**65.0 ± 1.2**	< 0.6	64.6 ± 1.2	< 0.6	62.8 ± 1.1	< 0.7
CRR-Net	64.8 ± 1.3	< 0.3	**64.8 ± 1.2**	< 0.3	**63.9 ± 1.4**	< 0.3

The test pairs are divided into three severity groups: mild ($\le 1\%$), moderate ($1\% - 4\%$), and severe ($> 4\%$), based on the percentage of foreground voxels with displacements greater than 10 mm. DSC: Dice similarity coefficient

**Table 6:** Quantitative evaluation of registration performance under varying deformation magnitudes on the ACDC dataset

Method	Mild		Moderate		Severe	
	DSC (%)	$\left| {{J_\phi }} \right| < 0$**(%)**	DSC (%)	$\left| {{J_\phi }} \right| < 0$**(%)**	DSC (%)	$\left| {{J_\phi }} \right| < 0$**(%)**
Initial	57.4 ± 9.3	-	58.4 ± 12.5	-	57.2 ± 6.3	-
SyN	80.7 ± 5.4	**0**	79.0 ± 6.9	**0**	80.1 ± 2.7	**0**
VoxelMorph	76.2 ± 7.4	< 0.07	73.9 ± 8.5	< 0.05	73.9 ± 5.7	< 0.02
TransMorph	79.0 ± 7.1	< 0.8	77.3 ± 8.1	< 0.8	76.7 ± 5.6	< 1
TransMatch	78.1 ± 7.3	< 0.1	76.8 ± 8.1	< 0.1	75.1 ± 5.1	< 0.1
PR++	81.2 ± 7.1	< 0.03	79.8 ± 7.0	< 0.02	81.5 ± 4.3	< 0.04
PIVIT	77.9 ± 8.7	< 0.004	75.8 ± 8.8	< 0.002	76.2 ± 6.2	< 0.003
Im2grid	72.4 ± 8.8	< 0.002	71.7 ± 9.3	< 0.004	73.5 ± 6.5	< 0.003
NICETrans	81.9 ± 5.5	< 0.1	80.2 ± 7.2	< 0.1	81.5 ± 4.4	< 0.1
ModeT	80.3 ± 6.7	< 0.007	79.4 ± 7.5	< 0.005	80.7 ± 3.9	< 0.01
RDP	**83.8 ± 4.0**	< 0.00005	82.7 ± 5.5	< 0.0002	82.9 ± 3.5	< 0.00003
CorrMLP	83.1 ± 5.0	< 0.1	82.3 ± 6.2	< 0.1	83.9 ± 2.8	< 0.1
CRR-Net	**83.8 ± 3.9**	< 0.04	**82.9 ± 4.8**	< 0.06	**84.0 ± 3.2**	< 0.06

The test pairs are divided into three severity groups: mild ($\le 1\%$), moderate ($1\%-4\%$), and severe ($> 4\%$), based on the percentage of foreground voxels with displacements greater than 10 mm. DSC: Dice similarity coefficient

### Ablation study

Ablation experiments were performed on different components of the CRRM, including the correlation layer, FRB, and attention, using the LPBA40 dataset. The experimental setup was as follows. The baseline model was defined as a model without these components, using vanilla convolution modules instead of the CRRM. Notably, the attention module consists of spatial and channel attention layers.

Table 7 presents the DSC scores for various component combinations. The baseline model achieved a DSC of 70.1% ± 2.2%. Introducing the correlation layer alone resulted in a 3.0% increase in DSC compared to the baseline model, indicating that computing correlation representations between fixed and moving features contributed to improved registration performance. Using the FRB alone produced a 3.2% increase in DSC, indicating that integrating super-resolution reconstruction at the feature level facilitated learning spatial correspondence between input images. When the correlation layer and FRB were combined, the performance improved by an additional 3.6% while yielding a smaller standard deviation. This demonstrates that reconstructing correlation representations between fixed and moving features is more effective for modeling spatial mappings than reconstructing the concatenated feature representations of fixed and moving images.

**Table 7:** Performance of different component combinations in the network

Correlation layer	FRB	Attention	DSC (%)	HD95 (mm)	ASSD (mm)	$\left| {{J_\phi }} \right| < 0$ (%)	FLOPs (G)
✗	✗	✗	70.1 ± 2.2	5.82 ± 0.55	1.76 ± 0.15	< 0.2	514.97
✓	✗	✗	73.0 ± 1.5	5.52 ± 0.48	1.60 ± 0.14	< 0.1	666.36
✗	✓	✗	73.3 ± 1.5	5.43 ± 0.47	1.56 ± 0.12	< 0.07	654.77
✗	✗	✓	70.5 ± 2.2	5.79 ± 0.53	1.74 ± 0.14	< 0.1	515.18
✓	✓	✗	73.5 ± 1.4	5.42 ± 0.47	1.54 ± 0.11	< 0.09	806.15
✓	✗	✓	73.1 ± 1.4	5.50 ± 0.47	1.58 ± 0.14	< 0.1	666.58
✗	✓	✓	72.7 ± 1.5	5.45 ± 0.45	1.57 ± 0.12	< 0.09	654.97
✓	✓	✓	73.7 ± 1.4	5.38 ± 0.46	1.53 ± 0.10	< 0.07	806.36

FRB: Feature reconstruction block; DSC: Dice similarity coefficient; ASSD: Average symmetric surface distance; HD95: 95% Hausdorff distance; FLOP: Floating-point operation

**Fig. 9:**
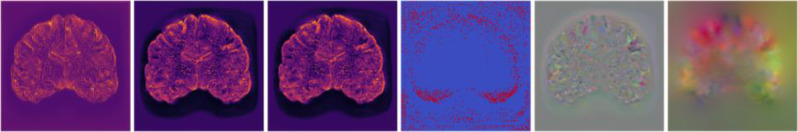
Feature maps processed by correlation reconstruction and refinement module and their corresponding visualizations. From left to right: the local correlation feature map, reconstructed feature map, attention-refined feature map, the difference map between the reconstructed and refined features, corresponding residual displacement field, and final deformation field

By contrast, introducing only the attention component did not achieve significant performance improvements. To evaluate the contribution of the attention component in the CRRM, three groups of experiments were designed: the combination of the correlation layer with the attention module, the combination of the FRB with the attention module, and the full combination of the correlation layer, FRB, and attention module. The results indicated that all three configurations yielded slight performance gains. To further investigate the impact of the attention component on the deformation field, full-resolution feature maps from different modules were analyzed (Fig. 9). These results suggested that the attention component effectively fine-tuned the deformation-field prediction, particularly in background regions where no real deformation occurred. Therefore, although the improvement in registration accuracy was minor, the attention component was retained in CRRM because of its ability to refine deformation-field prediction.

## Discussion

**Fig. 10:**
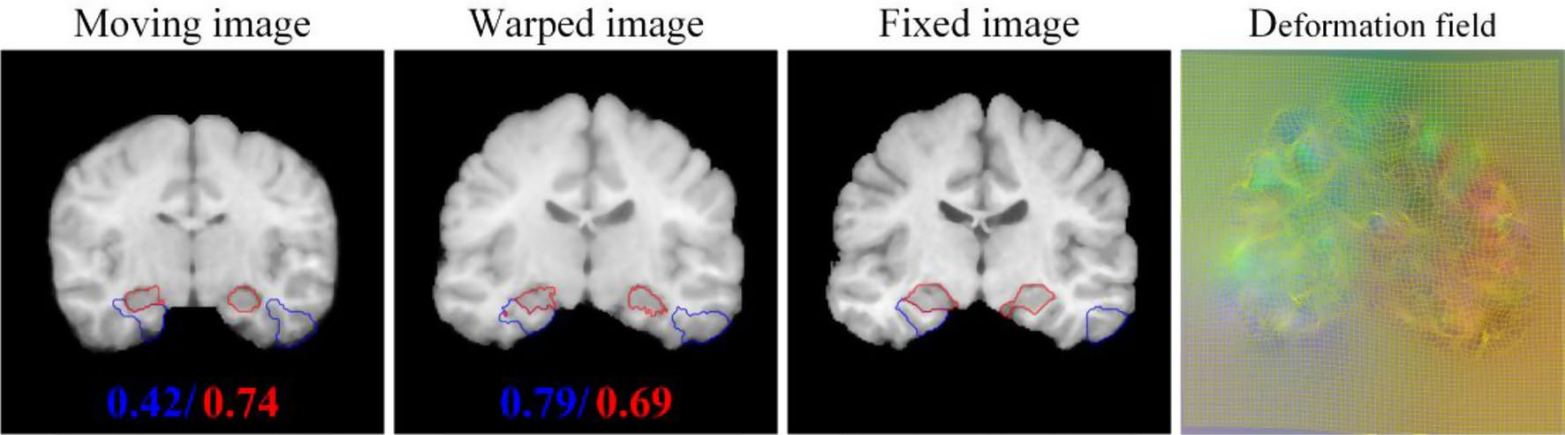
Representative failure case analysis. The red-outlined area represents the hippocampus, while the blue-outlined area represents the parahippocampal gyrus. The numerical values displayed at the bottom indicate the specific Dice similarity coefficient scores achieved for the corresponding regions of interest

In clinical practice, achieving precise alignment of moving images while maintaining diffeomorphic properties remains the primary objective of medical image registration. However, inter-patient registration often faces significant anatomical variability, making it challenging to balance rigorous alignment with preservation of the local topology. As illustrated by the deformation field composition in Fig. 4, the CRR-Net generates smooth global transformations at coarse scales; however, at finer scales, it inevitably introduces local grid folding to align highly complex anatomical details. Although the CRR-Net achieves superior accuracy in most cases, local over-optimization can lead to suboptimal outcomes. Figure 10 highlights an anomalous registration of the hippocampus: To forcibly align the severely displaced parahippocampal gyrus (blue outline), CRR-Net applied extreme deformations, successfully increasing its DSC from 0.42 to 0.79. However, this aggressive optimization warped the adjacent microstructures, causing severe folding and physical displacement in the hippocampus (red outline), where the DSC dropped from 0.74 to 0.69. Therefore, future research should prioritize exploration of smooth consistency constraints for local deformations to achieve a zero-folding rate.

Computational efficiency, which is dictated by the complexity and memory overhead, is another critical aspect of clinical viability. Although CRR-Net’s GPU inference speed outperforms mainstream methods such as CorrMLP, there remains room for optimization in high-resolution clinical deployments. The primary computational burden of the CRR-Net stems from the correlation layer and the FRB. As shown in Table 7, the correlation layer and FRB increase floating-point operations (FLOPs) by 151.39 G and 140.80 G, respectively, with a combined increase of 291.18 G. Analyzing the theoretical upper bounds of these modules is essential for lightweight design. For the input features ${F_1},{F_2} \in {\mathbb{R}^{C \times H \times W \times D}}$, upsampling factor $r$, kernel size $K$, and local neighborhood $k$, the theoretical FLOPs for the Correlation Layer are approximately ${\cal O}(C{k^3}HWD)$, with a memory footprint of ${\cal O}((C + {k^3})HWD)$. For the FRB, the theoretical FLOPs are ${\cal O}((3C + C/{r^3} + {k^3})C{K^3}HWD)$, and the memory footprint is ${\cal O}((2C + {k^3})HWD)$. Mathematically, both metrics scale linearly with the total number of voxels ($H \times W \times D$). Empirical analysis across resolutions from ${64^3}$ to ${256^3}$ (Table 8) confirmed that doubling the spatial resolution led to a cubic (${2^3}$) expansion of voxels, resulting in approximately an eightfold increase in FLOPs and inference memory. Notably, during training, the need to store intermediate activations for backpropagation significantly increased memory demands; at ${256^3}$, CRR-Net encountered out-of-memory errors. Thus, the development of memory-efficient architectures is essential for overcoming high-resolution bottlenecks.

**Table 8:** Computational complexity and memory consumption of correlation reconstruction and refinement network across various input resolutions

Resolution	Training memory (GB)	Inference memory (GB)	Floating-point operation (G)
64 × 64 × 64	1.24	0.38	43.00
128 × 128 × 128	9.12	3.04	344.05
256 × 256 × 256	-	19.36	2752.38
128 × 128 × 32	2.44	0.65	86.01
160 × 192 × 160	21.17	6.94	806.36

**Fig. 11:**
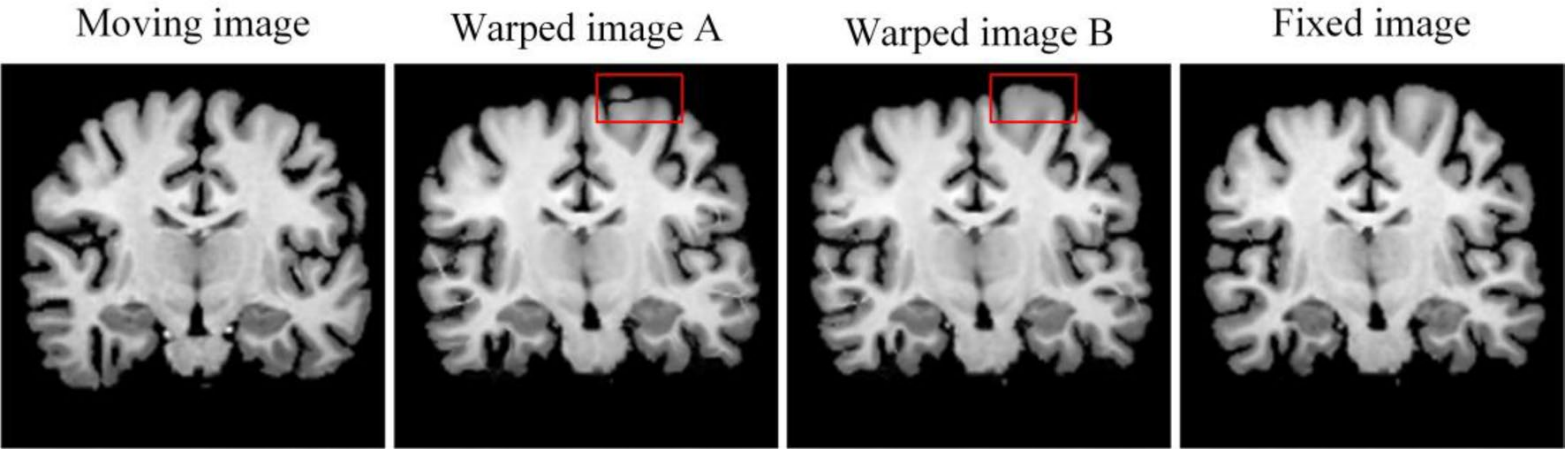
Qualitative comparison demonstrating the cross-dataset generalization capability on the Mindboggle dataset. Warped image a presents the inference results generated by the model trained exclusively on the LPBA40 dataset. Conversely, warped image B shows the optimal alignment achieved using weights trained specifically on the Mindboggle dataset. The red boxes highlight specific local anatomical regions for closer visual comparison of structural alignment details

Beyond topology and efficiency, cross-dataset generalization remains a common challenge in learning-based registration. Although CRR-Net excels within homogeneous data domains, its performance often degrades when deployed across heterogeneous datasets owing to inter-site domain shifts. As shown in Fig. 11, the model trained solely on the LPBA40 dataset exhibited a significant performance drop when applied to the unseen Mindboggle dataset. This degradation is largely attributable to the dependence of the network on specific training distributions, including MRI acquisition protocols, tissue intensity variations, and site-specific preprocessing. To reliably deploy the CRR-Net in multicenter clinical studies, we recommend fine-tuning the model using target-domain data. Furthermore, the current CRR-Net architecture was designed for monomodal registration and has not been extensively validated on multimodal data (e.g., CT to MRI) involving significant nonlinear intensity differences. Future work will focus on restructuring the feature-extraction paradigm to extend the super-resolution capabilities of CRR-Net to complex cross-modal tasks.

## Conclusions

This study demonstrated the effectiveness of CRR-Net for deformable medical image registration. The registration accuracy was enhanced by integrating super-resolution reconstruction into image registration at the feature level. This method facilitates finer structural alignment by leveraging enhanced high-resolution correlation representations and ensures consistent deformation-field predictions across coarse-to-fine scales. Extensive experiments on brain and cardiac image datasets showed that CRR-Net outperforms state-of-the-art deformable registration methods.

Despite these promising results, this study had several limitations. First, the CRR-Net was evaluated only for monomodal medical image registration, and its effectiveness in multimodal registration has not yet been fully validated. Second, although CRR-Net can handle many large local deformations, it may still encounter challenges in extreme cases involving substantial shear or large rotational motions. Third, the computational efficiency of processing correlation features at high resolutions requires further optimization to meet the requirements of real-world clinical applications.

Future work will further explore the applicability of this framework to multimodal image registration and investigate feature-level super-resolution reconstruction during the registration process. A more efficient strategy for correlation feature computation at super-resolution scales will also be developed to reduce computational overhead while preserving fine-grained structural alignment. In addition, the proposed super-resolution strategy has the potential to improve the fine-grained feature learning for various visual tasks with minimal adaptation.

## Data Availability

The LPBA40 MRI image datasets generated and analyzed in this study can be found in the ‘USC Stevens Neuroimaging and Informatics Institute’ repository, https://resource.loni.usc.edu/resources/atlases-downloads/. The Mindboggle MRI image datasets generated and analyzed in this study can be found in the ‘Mindboggle101_individuals’ repository, https://osf.io/yhkde/overview. The ACDC MRI image datasets generated and analyzed in this study can be found in the ‘Automated Cardiac Diagnosis Challenge (ACDC)’ repository, https://www.creatis.insa-lyon.fr/Challenge/acdc/index.html.

## Abbreviations

CRR-Net: Correlation reconstruction and refinement network
CRRM: Correlation reconstruction and refinement module
FRB: Feature reconstruction block
CNN: Convolutional neural network
ROIs: Regions of interest
DSC: Dice similarity coefficient
ASSD: Average symmetric surface distance
HD95: 95% Hausdorff distance
FLOP: Floating-point operation
MRI: Magnetic resonance imaging
